# Supplementary material for: Role of Cannabidiol for Improvement of the Quality of Life in Cancer Patients: Potential and Challenges
Source: Int J Mol Sci. 2022 Oct 26;23(21):12956. doi: 10.3390/ijms232112956 (PMC9654506; doi:10.3390/ijms232112956)
Supplement: Supplementary file 1 [file ijms-23-12956-s001.zip › ijms-1956645-supplementary.pdf]

Table S1. Common gene expression changes (logFC) in CBD treated glioma, neuroblastoma, and hepatocellular carcinoma cells.

Gene Symbol	Series GSE57978	Series GSE151512	Series GSE179661	
	Primary glioma	SK-N-BE(2)	MHCC97H	HepG2
MT1H	0.49	inf	13.78	12.84
MT1G	0.95	6.63	16.76	7.18
GDF15	2.25	5.30	3.06	4.77
HMOX1	1.53	2.84	1.25	5.49
AKNA	0.67	1.44	2.39	3.50
DDIT3	1.96	1.35	4.52	4.47
SESN2	1.32	1.16	2.64	4.31
TRIB3	1.84	1.03	1.94	4.42
GRB10	0.59	1.00	1.43	3.63
PLK3	0.58	0.85	0.90	4.39
ASNS	0.89	0.84	4.16	5.49
SLC3A2	1.30	0.81	2.17	4.63
UNC5B	1.43	0.69	3.73	4.99
FBXO32	1.26	0.67	4.40	3.68
SARS	0.76	0.65	1.52	1.68
ATF3	1.42	0.59	6.45	7.45
SQSTM1	0.92	0.56	1.44	4.08
HERPUD1	0.97	0.56	3.85	3.32
ADPRM	0.50	0.55	1.83	1.73
RBM24	1.02	0.54	1.55	4.73
UPP1	0.83	0.49	2.85	4.60
CARS	0.74	0.46	1.31	1.37
ANO10	0.81	0.45	1.51	1.00
CEBPG	0.86	0.42	1.57	1.40
GOT1	1.04	0.41	1.71	1.96
BCL6	0.64	0.40	0.78	1.55
ATF4	0.57	0.40	1.74	1.45
HBEGF	0.97	0.38	2.43	5.48
ZFP36	0.76	0.38	1.57	5.98
PPP1R15A	1.80	0.38	3.73	6.90
XBP1	0.99	0.31	1.32	1.42
SLC30A1	0.59	0.31	2.59	3.56
MARS	0.49	0.26	0.61	1.15
GADD45A	1.23	0.23	3.14	4.35
SNX25	0.71	0.22	0.25	0.63
TARS	0.47	0.18	0.68	1.67

EIF4EBP1	0.82	0.17	1.23	1.25
KDM7A	0.78	0.17	2.34	2.27
ZNF107	-0.68	-0.22	-1.42	-2.65
TMEM14A	-0.43	-0.33	-1.45	-1.19
TCF19	-0.94	-0.34	-0.72	-1.39
PIK3R1	-0.66	-0.47	-1.60	-2.95

Table S2. Gene expression changes in breast cancer cells (EVSA-T) treated with THC (Series GSE8502)

Gene Symbol	log(FC)
AA975768	2.20
ASNS	1.00
C1orf155	2.29
C6orf111	2.20
CALM3	-2.29
CCNA2	-1.14
CDC2	-2.23
CDKN1B	2.35
CPEB4	2.35
CYP51A1	-2.32
DHCR7	-2.46
DHFR	-2.38
DNAJA1	-2.32
EBP	-2.29
EIF1AP1	-2.17
EXOC2	1.20
FNTA	1.38
FTMT	2.32
GIPC1	-2.29
HERPUD1	1.00
HIST1H2AC	2.17
HSPA2	-2.17
HSPA8	-2.41
HSPH1	-1.07
IMPA1	2.38
IPO7	3.49
KCMF1	1.43
KIAA0101	-2.43
KIAA0146	-2.58
MCCC1	1.07
MCM3	-2.17

MCM6	-1.14
MYC	2.35
NP	-2.46
NUCB2	2.32
PABPC4	-2.29
PPIF	-2.23
RAB5A	1.43
RBM4	-2.23
RPS2	1.00
RRM2	-2.58
SEC31L1	2.20
SERINC1	1.07
SERPINB2	-2.49
SESN1	-2.51
SFRS3	-2.43
SHMT2	1.00
SLC3A2	1.68
TFRC	2.14
TGFB3	1.68
TMEM109	-2.32
TncRNA	2.58
TUBA2	-2.17
TUBA3	-2.26
TUBB	-2.29
UBADC1	-2.17
UHRF1	-2.23
USP3	1.49
VEGF	1.20
YY1AP1	2.20
ZFP36L1	2.29
ZNF265	2.20
ZNF451	1.32
ZUBR1	1.63
ZWINT	-1.43
